# Supplementary material for: Structural Studies of Pif1 Helicases from Thermophilic Bacteria
Source: Microorganisms. 2023 Feb 14;11(2):479. doi: 10.3390/microorganisms11020479 (PMC9964779; doi:10.3390/microorganisms11020479)
Supplement: Supplementary file 1 [file microorganisms-11-00479-s001.zip › microorganisms-2158528-supplementary.pdf]

Supplementary information

Structural studies of Pif1 helicases from thermophilic bacteria

Stéphane Réty [1,*], Yingzi Zhang [2], Wentong Fu [2], Shan Wang [2], Wei-Fei Chen [2] and Xu-Guang Xi [3,*]

1 Laboratoire de Biologie et Modelisation de la Cellule, Ecole Normale Superieure de Lyon, CNRS, UMR 5239, Inserm, U1293, Universite Claude Bernard Lyon 1, 46 allee d'Italie, F-69364 Lyon, France
2 State Key Laboratory of Crop Stress Biology in Arid Areas, College of Life Sciences, Northwest A&F University, Xianyang 712100, China
3 Laboratoire de Biologie et Pharmacologie Appliquée (LBPA), UMR8113 CNRS, ENS Paris-Saclay, Université Paris-Saclay, F-91190 Gif-sur-Yvette, France
* Correspondence: stephane.rety@ens-lyon.fr (S.R.); xxi01@ens-cachan.fr (X.-G.X.)

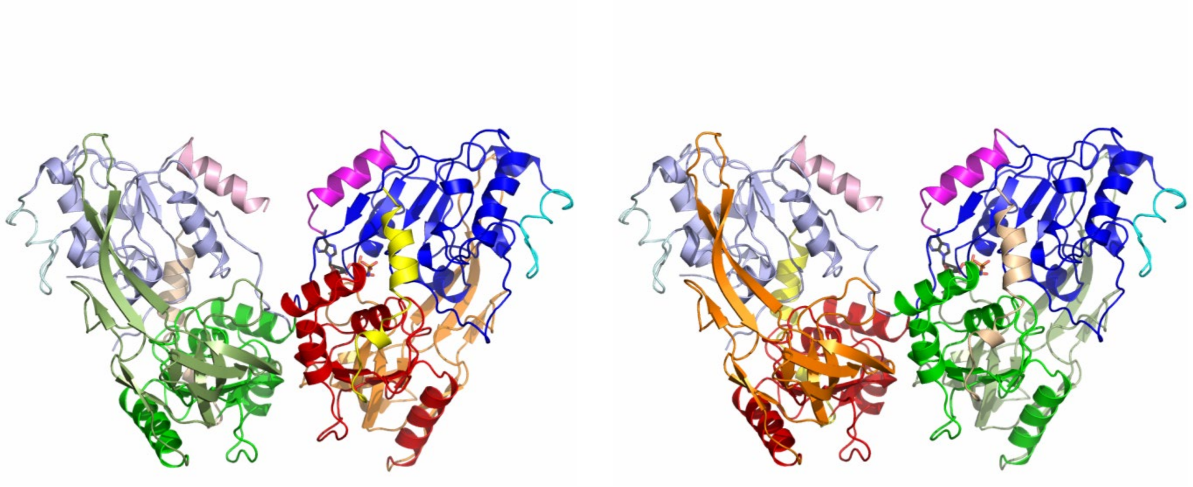

(**A**) (**B**)

Figure S1. Domain swapping in crystal of DdPif1-AMPPNP. **A.** The two molecules builT with no domain swapping. Molecule at the right is colored as in Figure 1A. **B.** The 2A and 2B domains have been swapped.

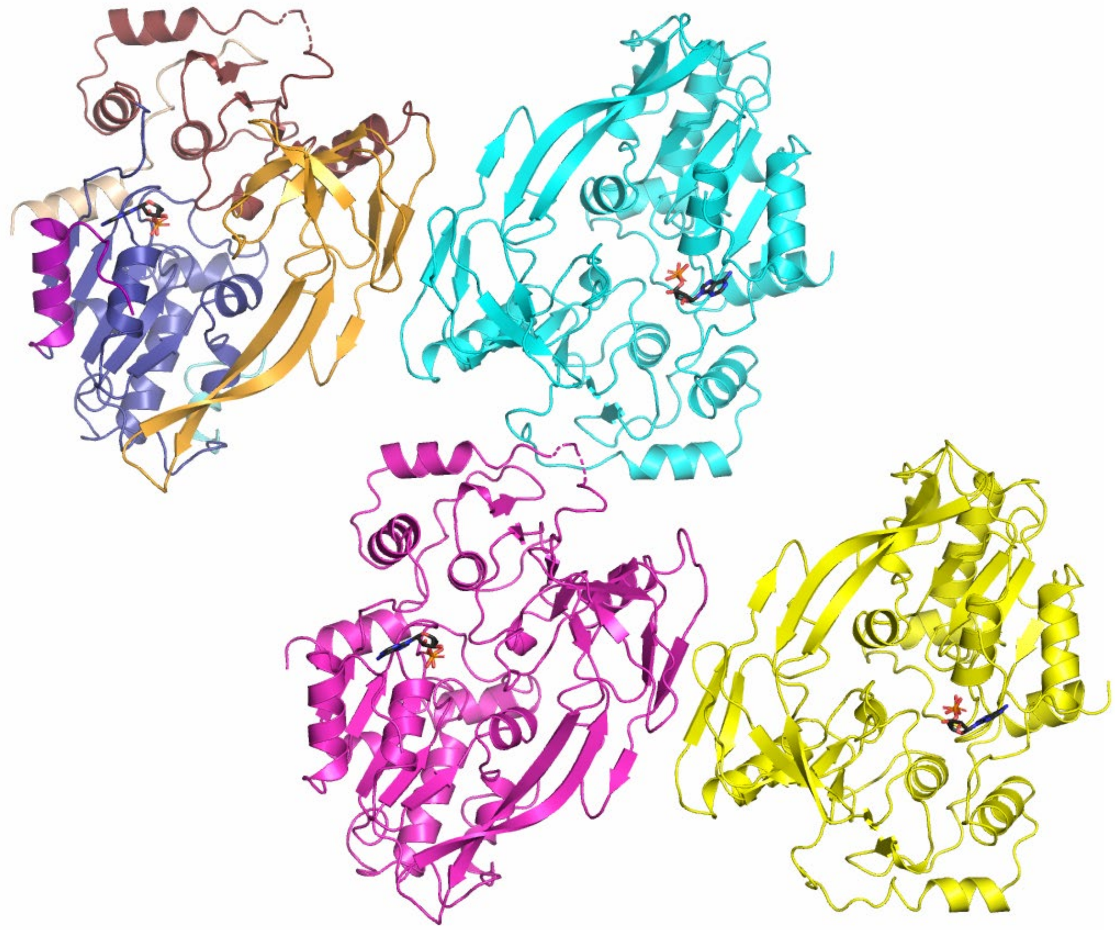

Figure S2. SSPif1-ADP crystal packing.

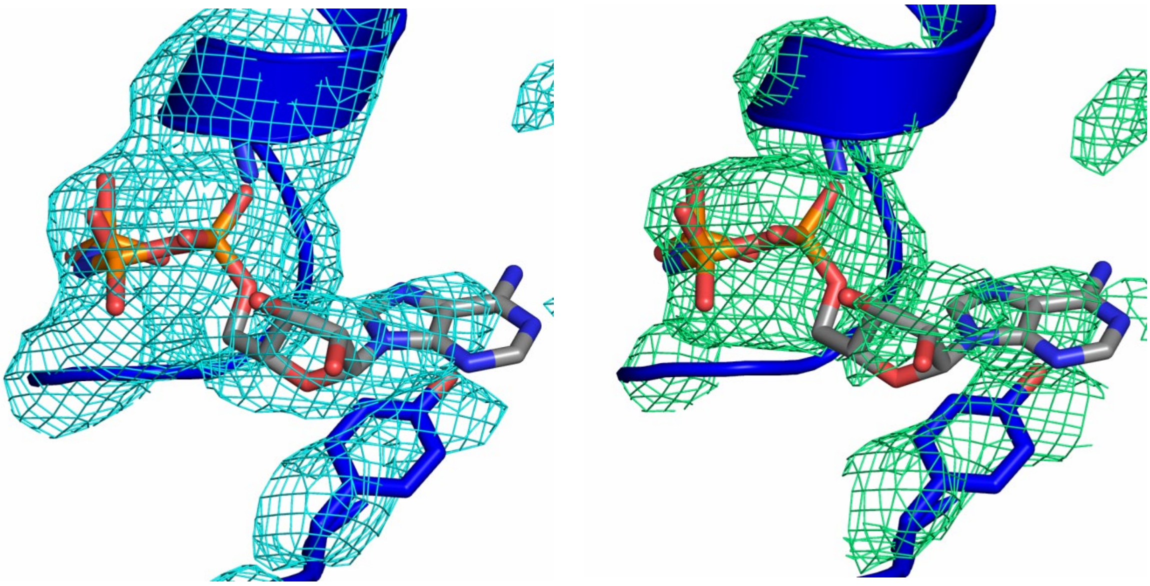

(**A**) (**B**)

Figure S3. Electron density map of DdPif1-AMPPNP complex around nucleotide (colored in grey). **A.** Final 2Fo-Fc map after refinement contoured à 1.5σ. **B.** Simulated annealing omit map contoured à 1.5σ.

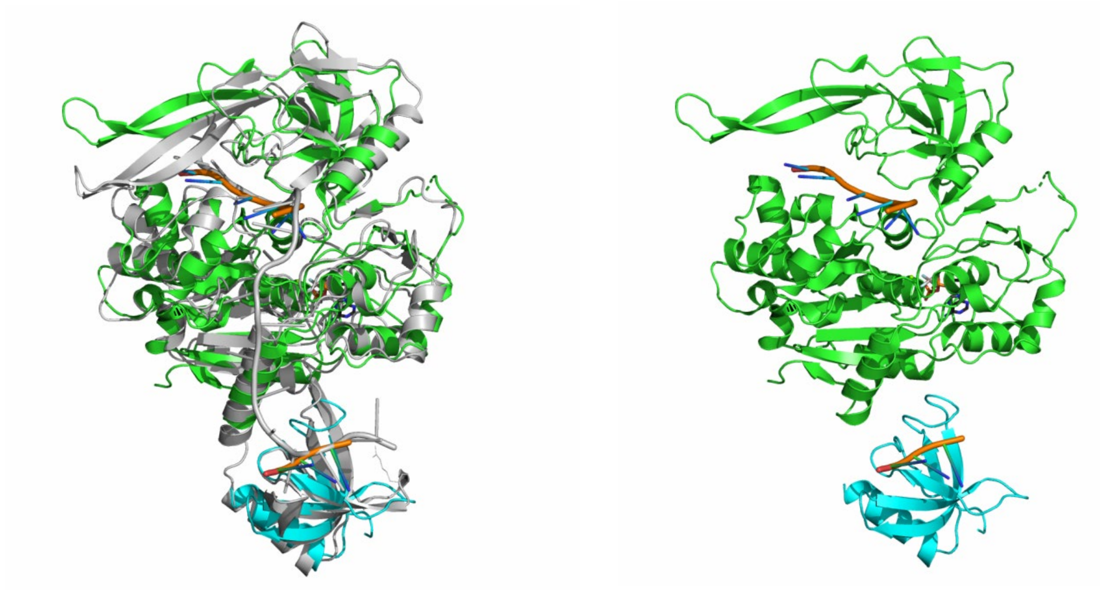

(**A**) (**B**)

Figure S4. DdPif1-ssDNA full length modeling and comparison with templates used. **A.** DdPif1 full length model is colored in grey and the templates BsPif1-ssDNA-ADP-Alf4 (PDB 5FHE) and WYL domain complexed with ssDNA (PDB 7U02) are shown in green and cyan respectively. **B.** For clarity, the templates are shown with no superimposed model.

Table S1. X-ray data collection and refinement statistics.

	**SsPif1 (PDB 8BNS)**	**DdPif1 apo (PDB 8BNV)**	**DdPif1 AMPPNP (PDB 8BNX)**
			**Data collection**
Wavelength (Å)	0.9778	0.9785	0.9785
Resolution range (Å)	110.1 - 3.24 (3.35 - 3.24)	37.64 - 2.86 (2.96 - 2.86)	71.9 - 3.12 (3.23 - 3.12)
Space group	$P2_1$	$C222_1$	$P2_12_12$
Unit cell			
a,b,c (Å)	113.138 129.29 113.68	88.76 147.05 78.37	143. 151.38 51.86
α,β,γ (°)	90 103.4 90	90 90 90	90 90 90
Unique reflections	49971 (266)	12140 (1198)	20930 (2068)
Multiplicity	3.4 (3.4)	6.4 (6.8)	6.5 (6.9)
Completeness (%)	91.6 (66.2)	99.49 (100.00)	99.87 (100.00)
Mean I/sigma(I)	5.2 (1.7)	15.11 (2.51)	10.49 (2.56)
Wilson B-factor (Å^2)	81.71	72.96	97.09
R-merge	0.150 (0.709)	0.08972 (0.7722)	0.1245 (0.7998)
CC1/2	0.996 (0.726)	0.996 (0.857)	0.991 (0.827)
**Refinement**			
R-work / R-free (%)	24.09 / 26.82	20.21 / 24.76	23.99 / 25.48
Number of non-hydrogen atoms	14316	3091	6733
macromolecules	14208	3077	6702
ligands	108	15	31
Protein residues	1732	381	830
RMS(bonds) (Å)	0.004	0.007	0.003
RMS(angles) (°)	1.01	0.95	0.68
Ramachandran			
favored (%)	99.59	99.47	99.51

allowed (%)	0.29	0.27	0.36
outliers (%)	0.12	0.27	0.12
Average B-factor (Å^2)	102.63	93.05	116.65
macromolecules	102.74	93.12	116.48
ligands	87.69	97.34	154.38

Statistics for the highest-resolution shell are shown in parentheses.

Table S2. SAXS data collection and processing.

	DdPif1 apo	
Structural parameters		
Guinier quality		
Data point	63	
*qR*g	0.408 - 1.296	
Correlation coefficient	0.996	
I(0) (cm^{-1}) [from Guinier]	4.41e03 +/- 9.4	
*R*g (Å) [from Guinier]	28.52 +/- 0.09	
*R*g (Å) [from *P*(*r*)]	28.04	
D_{max} (Å)	95.6	
Porod estimate (Å^3)	95855	
Molecular-mass determination		
Partial specific volume (cm^3.g^{-1})	0.74	
Contrast ($\Delta\rho \cdot 10^{10}$ cm^{-2})	2.82	
Molecular mass M_r [from *Vc*] (kDa)	46.5	
M_r [from *Vp*] (kDa)	49.9	
Calculated monomeric M_r from sequence (kDa)	47.0	
Data processing		
Primary data reduction	BioXTAS RAW	
Data processing	PRIMUS	
Ab initio analysis	DAMMIF	DENSS
Number of models	50	50
Model χ^2	1.05 +/- 0.01	0.23 +/- 0.18
Validation and averaging	DAMAVER	DENSS
NSD	0.67 +/- 0.15	0.86 +/- 0.12
Estimated resolution (Å)	25 +/- 2.6	31.4 +/- 5.2
Rigid-body modelling	DADIMODO	
Computation of model intensities	CRYSOL	
Model χ^2	1.224	
